# Editorial: MDS: new scientific and clinical developments

**DOI:** 10.3389/fonc.2025.1568681

**Published:** 2025-02-26

**Authors:** Sabine Blum, Argiris Symeonidis, Ulrich Germing

**Affiliations:** ^1^ Hematology Service and Central Laboratory of Hematology, Lausanne University Hospital and University of Lausanne, Lausanne, Switzerland; ^2^ Hematology Division, Department of Internal Medicine, University of Patras-School of Medicine, & Olympion General Clinic, Patras, Greece; ^3^ Department of Hematology, Oncology and Clinical Immunology, University Clinic, Heinrich-Heine-University Düsseldorf, Düsseldorf, Germany

**Keywords:** myelodyslastic syndromes, prognosis, diagnosis of MDS, flow cytometry in MDS, chelation, CHIP, CMML with SF3B1 mutation, treatment of MDS

The diagnostic approach and treatment of myelodysplastic syndromes or neoplasms (MDS or MDN) have progressed immensely over the past two decades. Initially a purely morphologic diagnosis supported by classical cytogenetics, has now evolved into a more delicately defined group of hematopoietic stem cell disorders, with the addition of many other diagnostic tools, such as more refined cytogenetics, molecular analyses, thorough histopathology/immunohistochemistry and flow cytometry, aimed at more precisely classifying MDS subtypes, for an integrated diagnostic approach and risk factor estimation. This Research Topic of Frontiers in Oncology is dedicated to the new scientific and clinical developments in the field of MDS and aspires to become a useful source of information for all scientists working in this field. The original work from Nachtkamp et al. explains the discrepancy and concordance of results between cytology and histomorphology and shows for which type of information cytology is preferable and for which type histology is reliable The review paper by Oster and Mittelman discusses how to approach the diagnosis of this complex group of neoplasms with fewer interventional methods, and in line with this approach, Verigou et al. describe the role of multiparametric flow cytometry in their review, in light of the molecular era here. In addition to classical cytogenetic analysis and NGS, the role of microRNA dysregulation is analyzed in the review by Micheva and Atanasova. Over the decades, different prognostic scores for MDS have been developed, such as the IPSS; the WPSS; the IPSS-R and more recently the IPSS-M, to help estimate survival and transformation rates to acute myeloid leukemia (1–4). In their original work, Zamanillo et al. demonstrate a retrospective validation of the M-IPSS in their MDS patient cohort here.

We still do not know exactly how and why MDS develops, but the role of an intact immune system in preventing the development of MDS or delaying its evolution appears to be crucial and is supported by a higher incidence of MDS in patients suffering from autoimmune or over-immune diseases (5). Janssen et al. explain the role of monocytes and thrombomodulin expression on monocytes in the pathogenesis of MDS here. An established risk factor for the development of MDS is the preexistence of clonal hematopoiesis of indeterminate potential (CHIP) (6). In a comprehensive review Cacic et al. present all the relevant aspects for the medical counseling of patients diagnosed with CHIP, as there is not much that can be done to prevent progression to MDS, except to avoid chemotherapy whenever possible. Despite the fact that in the latest version of the WHO classification, published in 2022, several new MDS subtypes have been defined in a more granular way, many patients cannot be precisely classified according to this classification (7). In their original work, Xicoy et al. describe the hybrid entity of chronic myelomonocytic leukemia with ring sideroblasts and SF3B1 mutation, which is strikingly similar to the entity of MDS with low blasts and SF3B1 mutation.

Many patients with MDS are still treated with best supportive care only, even though in the last decades new treatment approaches for higher-risk MDS and specific, targeted treatments for some forms of lower-risk MDS, especially for MDS with low bone marrow blasts (LB) and SF3B1 mutation and for MDS with LB and 5q deletion have become available. However, for the majority of MDS subtypes, no specific treatment is available, and patients only receive supportive red blood cell and platelet transfusions (8, 9). In these patients, very often iatrogenic iron overload occurs, with a need for iron chelation therapy. Iron homeostasis and its regulation are complex, and new regulators such as the “master regulator” hepcidin and its regulator erythroferrone have been discovered in the last decades (10). This mini review by Abba et al. focuses on the role of erythroferrone in MDS.

Molecular diagnostics allow us to choose tailored treatment approaches in some MDS subgroups, such as SF3B1 mutated MDS with low blasts. In this subgroup, which has the best prognosis of all MDS subtypes, both erythropoietin and luspatercept are approved as treatment options (8, 11). These drugs are normally used sequentially, but in this original article, Jonasova et al. describe their real-world experience with the combination of both drugs in lower-risk MDS.

In patients who do not undergo allogeneic stem cell transplantation, we know that intensive chemotherapy does not lead to an increase in overall survival in the majority of cases (12). Since the publication of the phase 3 trial of 5-azacytidine (AZA), versus the conventional care regimen (intensive chemotherapy, low-dose cytarabine or best supportive care), known as the AZA-001 trial, treatment of higher-risk MDS with AZA is considered the gold standard (13). Although response rates and survival have been shown to be better with AZA, more than half of the treated patients do not respond to this treatment, and on the contrary may suffer from side effects of the drug, mainly due to the worsening of pre-existing cytopenias. To date, we do not have tools available that would allow us to predict response and spare the non-responders a treatment trial. In this Research Topic, González et al. provide data on molecular profiling, that may help to predict future responders to AZA treatment in this original paper. In elderly patients who progress from MDS to AML, the combination of AZA with Venetoclax (VEN+ AZA) is now the standard of care (14). However, the majority of patients will experience a relapse and will ultimately die from their disease. Deciphering the resistance mechanisms that lead to relapse will help to further prolong the survival of this patient population in the future. Finally, Bašovà et al. contribute to this Research Topic with a mouse model of VEN+ AZA resistance, which they describe in their original paper. The authors also tested drug combinations to overcome this resistance.

This Research Topic touches on major clinical problems about the diagnosis, prognosis, and treatment of patients with MDS and will hopefully inspire both clinicians and scientists.

We hope that the readers will enjoy reading this gallery of manuscripts and will enrich their knowledge of both, basic research and clinical practice.
